# Quality of Life in Patients With Rectal Resections and End-to-End Primary Anastomosis Using a Standardized Perioperative Pathway

**DOI:** 10.3389/fsurg.2021.789251

**Published:** 2022-01-07

**Authors:** Jonas Herzberg, Shahram Khadem, Valentin Begemann, Tim Strate, Human Honarpisheh, Salman Yousuf Guraya

**Affiliations:** ^1^Department of Surgery—Krankenhaus Reinbek St. Adolf-Stift, Reinbek, Germany; ^2^Clinical Sciences Department, College of Medicine, University of Sharjah, Sharjah, United Arab Emirates

**Keywords:** rectal resection, PROM (patient reported outcome measures), quality of life, colorectal surgery, anastomosis

## Abstract

**Objectives:** Lower rectal resection is associated with a high rate of postoperative complications and, therefore, adversely impacts the postoperative health-related quality of life (QoL). Though sporadically practiced in different centers, there is no standard perioperative protocol for the management of patients with rectal growths. The aim of this analysis is to evaluate the patient-reported outcomes after low rectal resections followed by an end-to-end-reconstruction and temporary covering ileostomy using a multidisciplinary fail-safe-concept.

**Methods:** Between 2015 and 2020, we evaluated patient reported outcomes after open and laparoscopic rectal resections with end-to-end reconstruction with a primary straight anastomosis using a standardized perioperative pathway All patients with stoma were excluded from the study. The data for the QoL of patients was collected using the established Low Anterior Resection Syndrome (LARS)-score and the EORTC-C30 and CR-29 questionnaires at a single postoperative timepoint.

**Results:** We recruited 78 stoma-free patients for this analysis. Of 78 patients included in the study, 87.2% were operated laparoscopically and the mean global health status was 67.95 points, while a major LARS was detected in 48 (61.5%) patients. No anastomotic leakage (AL) occurred within the study cohort. There was no significant change in the LARS-score or the global health status depending on the follow-up-period.

**Conclusion:** This study shows that good QoL and functional outcomes with no AL are achievable following end-to-end straight anastomosis using a standardized perioperative surgical fail-safe protocol procedure.

## Introduction

Colorectal cancer is the third leading cancer worldwide and the second leading cause of cancer-related deaths with an estimated 1.8 million new cases per year and 881,000 deaths worldwide (1, 2). Surgical therapy remains the gold standard for rectal growths and the outcomes of surgical treatment for rectal pathologies primarily depend on the location and stage of the tumor, the perioperative surgical protocol and the surgical technique (3). The most reported adverse mid-term-consequence of low anterior resection (LAR) is a deranged bowel function, often referred as “low anterior resection syndrome” (LARS) (4). The manifestations of LARS are far ranging; fecal incontinence, urgency, evacuatory and sexual dysfunctions, abnormal bowel frequency. As evident, LARS carry a direct impact on the quality of life (QoL) after rectal surgery (5). QoL, the individual's state of wellbeing, is deeply influenced by illness and treatment, especially in cancer patients (6).

In the recent years, the health-related quality of life (HRQOL) has been recognized as a mandatory requirement for the approval process of new anticancer drugs by the European Medicines Agency (7). Additionally, during the routine management of patients with a range of elements, the HRQOL has been embedded as a part of the patient-reported outcomes. LAR with primary anastomosis carries a high risk for adverse postoperative patient-reported outcomes due to a rise in postoperative complications such as anastomotic leakage (AL), sepsis and delayed bowel functions (8). Moreover, sphincter-preserving rectal surgery often leads to autonomic nerve damage with its associated functional disorders. Following LAR for the rectal cancer, defecation disorders have been reported in 41% (9), sexual dysfuction in 64% (10) and urinary dysfunctions in 50% patients (10). Such alarming rates of complications following LAR adversely affect the patient's psychosocial health status and the HRQOL.

Different tools for the assessment of patient-reported outcomes and HRQOL for rectal resections have been tested and validated. For a short-term evaluation, a popular instrument is the time-tested 5-item LARS score, which includes questions for incontinence for flatus or stool, the frequency of bowel movement, incomplete defecation, or urgency (11–13). This simple tool, available in different languages (13–15), focuses on the postoperative defecation disorders following rectal resections and correlates well with other QoL questionnaires (16).

In addition to the LARS questionnaire, the European Organization for Research and Treatment of Cancer (EORTC) has developed a well-evaluated 30-item core questionnaire (QLQ-C30), which investigates the general QoL with additional procedure-related instruments such as the questionnaire for the colorectal cancer (QLQ-C29) (17).

A range of remedial steps have been taken to prevent the dreadful long-term functional complications following rectal resections for cancerous growths. To reduce the rate of autonomic nerve complications, the intra-operative autonomic nerve preservation has been successfully established (18–21). This mandates the use of laparoscopic or robotic surgery in high-volume centers by experienced colorectal surgeons. The reservoir functions of the rectum is lost following the resection for the rectum along with its ampulla (22). In order to prevent a high stool frequency, the bowel reconstruction could be performed using a J-pouch (23), a coloplasty (24) or side-to-end-reconstruction (22, 25). This surgical step might reduce the frequency of defecation; however, the placement of sutures or stapling lines can possibly lead to an increased leakage rate. Following these beneficial observations in the literature, the German guidelines for colorectal cancer surgery recommend the use of a reservoir building reconstruction such as pouch or end-to-side-reconstruction, wherever possible (26).

Despite an escalating rise in the rates of complications and poor QoL after rectal surgery, unfortunately there is no standard peri-operative management protocol that can mitigate these risks. Though literature has shown the QoL related outcomes of patients following rectal surgery using cross-sectional study designs, there is a limited data about the reference population or pretreatment guidelines (27, 28).

The aim of this study is to evaluate the patient-reported outcomes after low rectal resections and end-to-end-reconstruction for benign and malignant rectal lesions using a multidisciplinary fail-safe-protocol. We used the EORTC-C30, C29 questionnaires and the LARS-score for the assessment of the QoL after rectal resections, which provide an insight into the efficacy and safety profile of the fail-safe peri-operative protocol.

## Materials and Methods

### Study Design

In this study, we prospectively included all patients who underwent open or laparoscopic rectal resections with end-to-end anastomosis at Reinbek St. Adolf-Stift Hospital Germany to a colorectal surgical database. Between January 2015 and December 2020, patients with a tumor localized ≤ 8 cm from the anal verge were treated by rectal resections and end-to-end primary anastomosis. The hallmark of our management plan was the multi-modal fail-safe protocol, which included a standard surgical technique for tension-free anastomosis was adapted in the fail-safe approach. All patients with rectal resections and primary end-to-end-anastomosis had an ileostomy, which were closed after the completion of adjuvant therapy soonest 6 weeks after the primary procedure. Patients who still had ileostomies at the time of conducting this study were excluded from the cohort. The patients' median follow-up period was 1 year.

After obtaining the ethical approval, the patients' medical records were extracted from the prospective clinical database according to the established inclusion criteria. Later, all recruited patients were invited to participate in this research. All patients gave their written informed consent to participate in this study. The EORTC-C30, C29 questionnaires and the LARS-scoring tool were posted to all patients by registered post at a single timepoint. The data presented in this study are reported in concordance with the STrengthening the Reporting of OBservational studies in Epidemiology (STROBE) criteria (29). This trail was registered in the German Clinical Trial Register (DRKS00022492, date of registration: 10/20/2020).

### Perioperative Fail-Safe Protocol

All patients suspected with rectal cancers were staged according to the German guidelines for colorectal cancer (26). Depending on the preoperative staging and according to the decision of the interdisciplinary tumor board, patients were treated by neoadjuvant radiotherapy, chemoradiotherapy or primary surgery. Patients with benign rectal lesions were not discussed during the interdisciplinary tumor board meetings. In case of severe diverticulitis, extended resections were performed.

**Figure 1:**
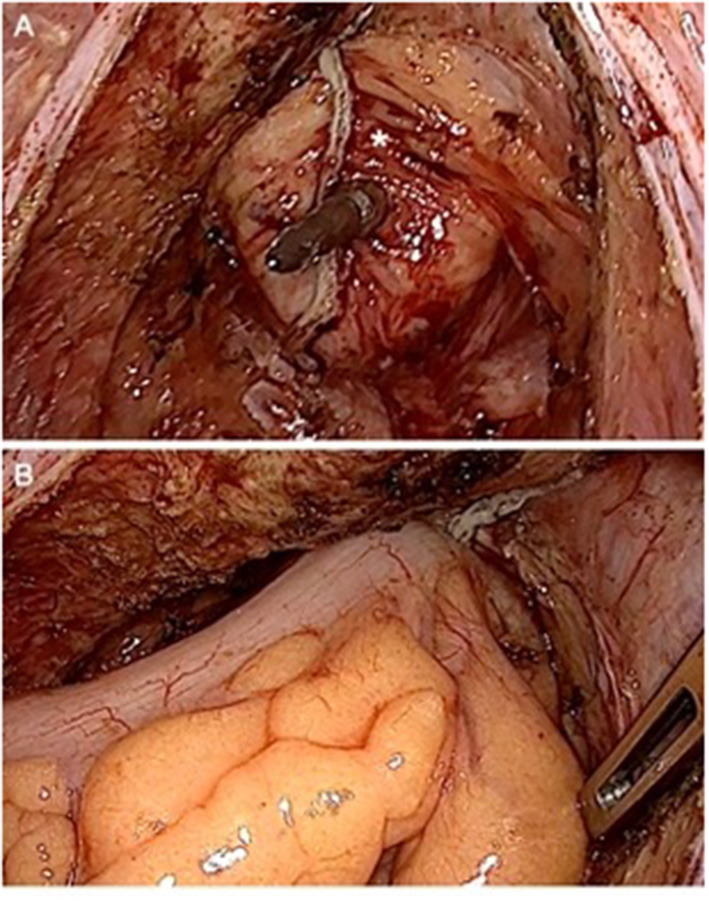
Performing an end-to-end reconstruction after low rectal resection. **(A)** Rectal stump (*) still covered with fatty tissue to ensure perfusion with the spine of the stapler is piercing near the previous stapling line. **(B)** Compression after joining both ends to flatten fatty tissue before releasing the stapling device.

**Figure 2:**
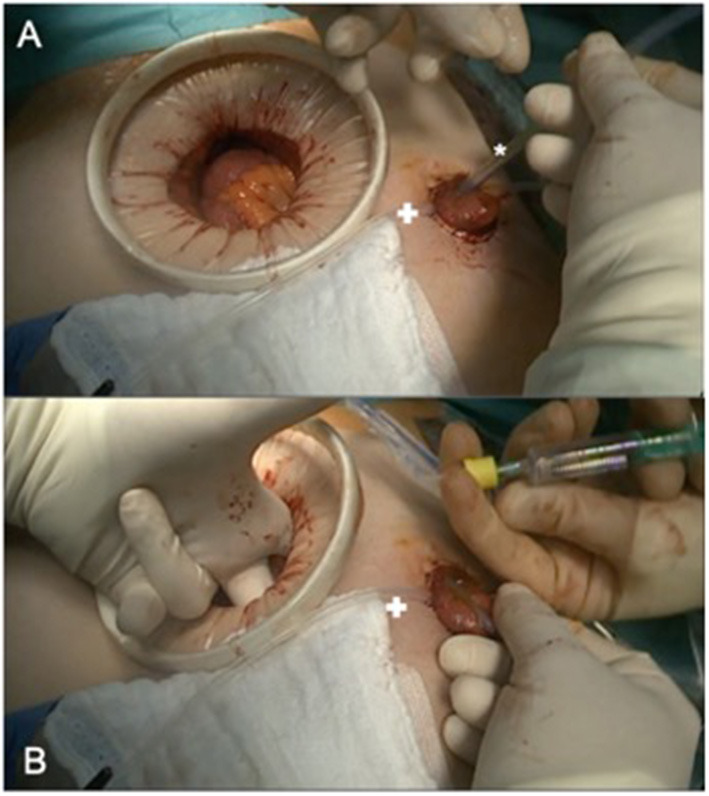
Intraoperative colonic irrigation via ileostomy. **(A)** Placing the catheter (*) in the efferent loop (+ marking a loop, fixing the diverting stoma until fixation is completed. **(B)** Blocking the catheter under manual controll before starting the antegrade colonic irrigation.

All patients were treated according to the fail-safe-protocol with a preoperative mechanical bowel preparation using 2l of Endofalk®. No additional oral antibiotic decontamination was deemed necessary. In case of primary open procedures, epidural anesthesia was established. A single-shot-antibiotic was given intravenously perioperatively using 500 mg metronidazole and 1500 mg cefuroxime. After performing an end-to-end stapling anastomosis (Figure 1), a drainage was placed in the pelvis near the anastomosis and a diverting ileostomy was performed. In addition, an on-table-lavage via the efferent loop of the ileostomy was used to reduce the fecal load near the anastomosis (Figure 2). Three days after surgery, an endoscopic evaluation of the anastomosis was routinely performed. The diverting ileostomy was reversed after the completion of adjuvant therapy, if needed, at least 6 weeks after surgery and after performing colonoscopy for the evidence of intact anastomosis.

Postoperative complications were graded in accordance to the established Dindo-Clavien grading system, where all complications graded 3b and above are considered as major complications (30).

### Patient Reported Outcomes

The EORTC-C30, C29 questionnaires and the LARS-scoring tool were used to measure the patient-reported outcomes and postoperative functional results in colorectal surgery.

#### LARS Score

The LARS score is a well-established simple scoring tool for the evaluation of bowel function after rectal resections. This tool assesses postoperative incontinence for flatus and liquid stool, frequency of bowel movement, incomplete defecation and urgency (13, 31). The final score in the LARS score ranges from 0 to 42; a score below 20 points indicates an absence of LARS, 21–29 is interpreted as a minor LARS and 29 up to 42 as a major LARS.

#### EORTC-C30 and C29

The EORTC-C30 measures the QoL regarding a global health status and contains five functional and nine symptoms scales. Depending on the responses by patients, according to the EORTC manual, a score ranging from 0 to 100 is calculated (17). Pursuant to the EORTC scoring system, a high score for the function or global health status indicates a better HRQOL; whereas a higher symptom scale means a greater burden by the scored symptom (17).

This tool was specified by an organ related module for colorectal malignancy with further 38 specific questions (QLQ-CR29) (32). The EORTC-C29 questionnaire was also scored following the published EORTC scoring manual (32). Some questions are focused on stoma-related issues which are excluded for non-stoma-patients. All data are compared to evaluate reference values (33). Also this combined EORTC-questionnaire with 68 items in total is very long, it provides a conclusive impression about the individual quality of life including organ-specific complications.

### Statistical Analysis

All data was analyzed using SPSS 25.0 (IBM Corp, Ammonk, NY). The Chi-Square-test was used to compare categorical variables, and in case of <25 cases, the Fisher's exact test was used. In case of more than two groups, Kruskal-Wallis-test was performed. Continuous variables are presented as means and standard deviation as exemplified by the EORTC (17). For inter-group-evaluation, according to the EORTC and previous studies differences, were rated 5–10 as small difference, 10–20 moderate and more than 20 as a large difference (5). The Mann-Whitney U-test was used for inter-group comparison. A *p*-value of < 0.05 was defined statistically significant.

## Results

During the study timeframe, 1.987 colorectal surgical procedures were performed in our center. This included 153 patients with rectal resections and with primary anastomosis. Twelve patients (7.8%) died during the follow-up-period and 12 patients (7.8%) were lost to follow-up. The questionnaire including the written consent form was sent to all remaining 129 patients. Seventy-eight returned the completed questionnaire (60.5%). Overall, there were 45 male (57.7%) and 33 females (42.3%) with a mean age of 65.64 ± 12.24 years. As many as 20.5% of the surgical procedures were performed for benign rectal lesions such as extended diverticulitis or large polyp of the rectum and 79.5% procedures were performed for malignant rectal growths. In this study cohort, no AL was reported. At the same time, 67 patients (85.9%) had an uneventfull recovery. Major complications requiring intervention under general anesthesia (Dindo-Clavien > 3b) were found in four patients (5.1%). These included one stoma revision, one subcutaneous hematoma, one uretheral obstruction without injury of the urethera, and a case of splenic bleeding which was treated by splenectomy.

In our series, the majority (87.2% were operated laparoscopically with no postoperative AL. The mean time to follow-up were 19.5 months. Major LARS was detected in 48 (61.5%) patients. There was no significant correlation between the time to follow-up- and the rate of major LARS following the end-to-end rectal reconstruction. Patients' characteristics did not differ significantly according to no/minor LARS or major LARS are shown in Table 1. The duration of surgery was significantly longer in patients with major LARS (285.15 min ± 77.05 min) than in patients without major LARS (228.50 min ± 71.37 min, *p*-value 0.001).

**Table 1:** Patient characteristics according to LARS/Major LARS.

	**Total** **(***n*** = 78)**	**No/minor LARS** **(***n*** = 30)**	**Major LARS** **(***n*** = 48)**	***P***
Age [years] (mean ± SD)	65.64 ± 12.24	65.60 ± 12.71	65.67 ± 12.08	NS^c^
BMI (mean ± SD)	27.51 ± 8.89	25.94 ± 3.83	28.49 ± 10.86	NS^c^
Sex, n (%)				
Male	45 (57.7)	17 (56.7)	28 (58.3)	NS^b^
Female	33 (42.3)	13 (43.3)	20 (41.7)	NS^b^
ASA, n (%)				
I	4 (5.1)	3 (10.0)	1 (2.1)	NS^b^
II	54 (69.2)	20 (66.7)	34 (70.8)	NS^b^
III	20 (25.6)	7 (23.3)	13 (27.1)	NS^b^
Procedure, n (%)				
Laparoscopic, n (%)	68 (87.2)	25 (83.3)	43 (89.6)	NS^b^
Open	8 (10.3)	4 (13.3)	4 (8.3)	NS^b^
Conversion	2 (2.6)	1 (3.3)	1 (2.1)	NS^b^
Length of surgery [min] (mean ± SD)	263.36 ± 79.45	228.50 ± 71.37	285.15 ± 77.05	0.001
Time to follow-up [months] (mean ± SD)	19.50 ± 16.86	22.07 ± 17.77	17.90 ± 16.24	NS^c^
Global Health status (mean ± SD)	67.95 ± 20.37	75.83 ± 18.49	63.02 ± 20.11	0.003^c^
Major complication (DC > 3b), n (%)	4 (5.1)	4 (5.1)	0 (0)	0.019^b^
Dignity, n (%)				
Benign	16 (20.5)	8 (26.7)	8 (16.7)	NS^b^
Malign	62 (79.5)	22 (73.3)	40 (83.3)	NS^b^
	*n* = 62	*n* = 22	*n* = 40	
N+ (%)^a^	24 (38.70)^a^	9 (40.9)	15 (37.5)	NS^b^
T3/4 (%)^a^	29 (46.8)^a^	10 (45.5)	19 (47.5)	NS^b^
R0, n (%)^a^	61 (98.4)^a^	22 (100.0)	39 (97.5)	NS^b^

*DC, Dindo-Clavien classification*.

a *Including only cases with malignancy (n = 62)*.

b *Fisher exact test*.

c *Man-Whitney-U-test*.

**Figure 3:**
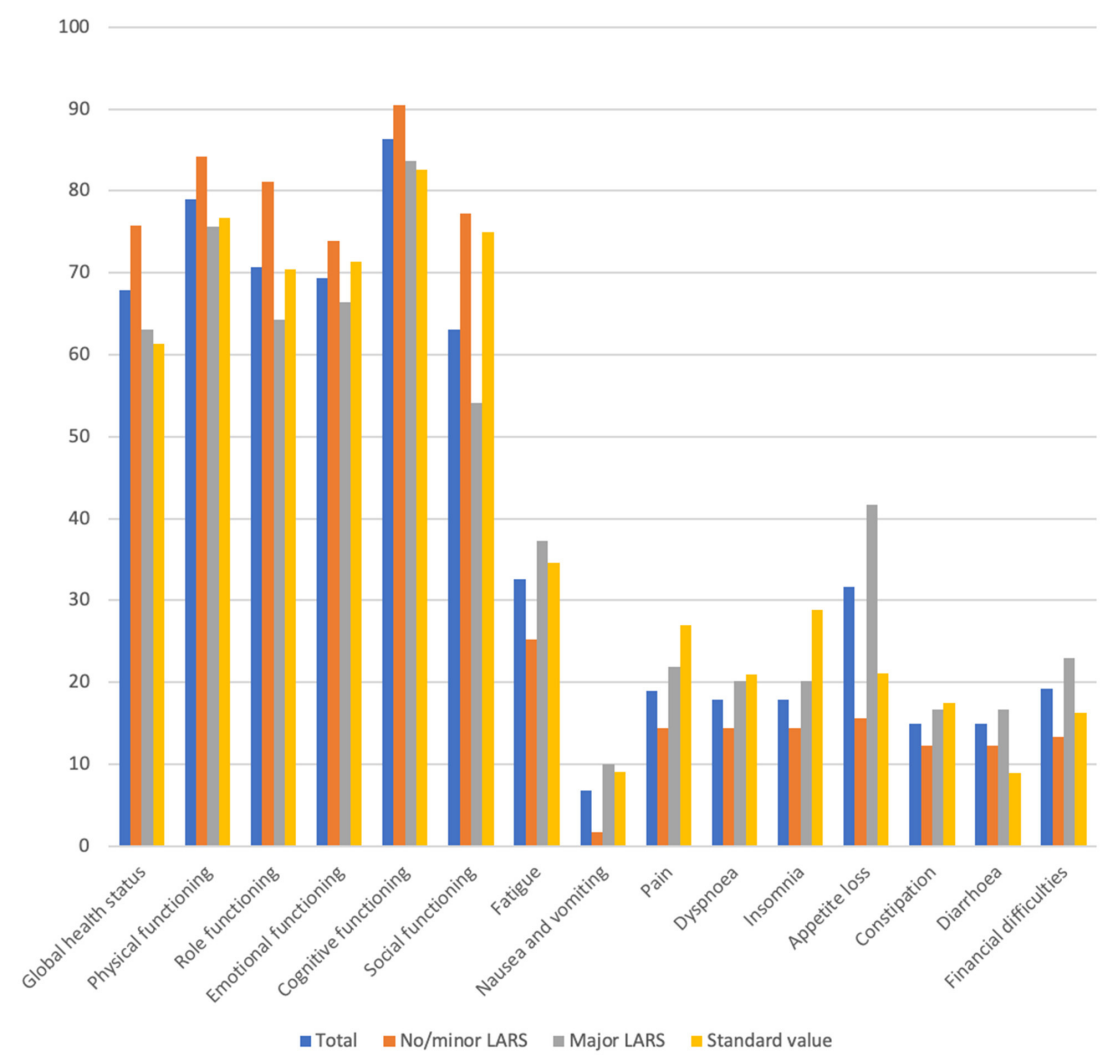
Global health relatated quality of life (mean). According to the EORTC-scoring manual a high score in fuctional scales represents a high functional level wehreas a high score in symptom scales correlate with a high level of symptoms.

Using the EORTC-C30 questionnaire, the mean global health status score in our study cohort was 67.95 points. This score differed significantly between the major LARS and no/minor LARS groups (63.02 vs. 75.83, *p* = 0.003). A significant difference was observed between these groups in terms of physical functioning (*p* = 0.031), role functioning (p=0.012), social functioning (*p* = 0.002), and nausea and vomiting (*p* = 0.017) (Figure 3). There were no significant differences in patients' characteristics for a low or high global health status (Table 2).

**Table 2:** Patient characteristics according to the global health status.

	**Global health status > 65** **(***n*** = 51)**	**Global health status <65** **(***n*** = 27)**	***p*** **-value**
Age [years] (mean ± SD)	65.06 ± 11.10	66.74 ± 14.32	NS^c^
BMI (mean± SD)	28.21 ± 10.23	26.18 ± 5.50	NS^c^
Sex, n (%)			
Male	30 (58.8)	15 (55.6)	NS^b^
Female	21 (41.2)	12 (44.4)	NS^b^
ASA, n (%)			
I	3 (5.9)	1 (3.7)	NS^b^
II	35 (68.6)	19 (70.4)	NS^b^
III	13 (25.5)	7 (25.9)	NS^b^
Technique, n (%)			
Laparoscopic	44 (86.3)	24 (88.9)	NS^b^
Open	5 (9.8)	3 (11.1)	NS^b^
Conversion	2 (3.9)	0 (0.0)	NS^b^
Length of surgery	254.04 ± 74.65	280.96 ± 86.51	NS^c^
[min] (mean ± SD)			
Time to follow-up	19.02 ± 17.18	20.41 ± 16.52	NS^c^
[month] (mean ± SD)			
Major LARS, n (%)	28 (54.9)	20 (74.1)	NS^b^
Major complication (DC > 3b), n (%)	2 (3.9)	2 (7.4)	NS^b^
Dignity, n (%)			
Benign	12 (23.5)	4 (14.8)	NS^b^
Malignant	39 (76.5)	23 (85.2)	NS^b^
	*n* = 39	*n* = 23	
N+ (%)^a^	18 (46.2)	6 (26.1)	NS^b^
T3/4 (%)^a^	19 (48.7)	10 (43.5)	NS^b^
R0 (%)^a^	38 (97.4)	23 (100.0)	NS^b^

a *Including only cases with malignancy (n = 62)*.

b *Fisher exact test*.

c *Man-Whitney-U-test*.

**Figure 4:**
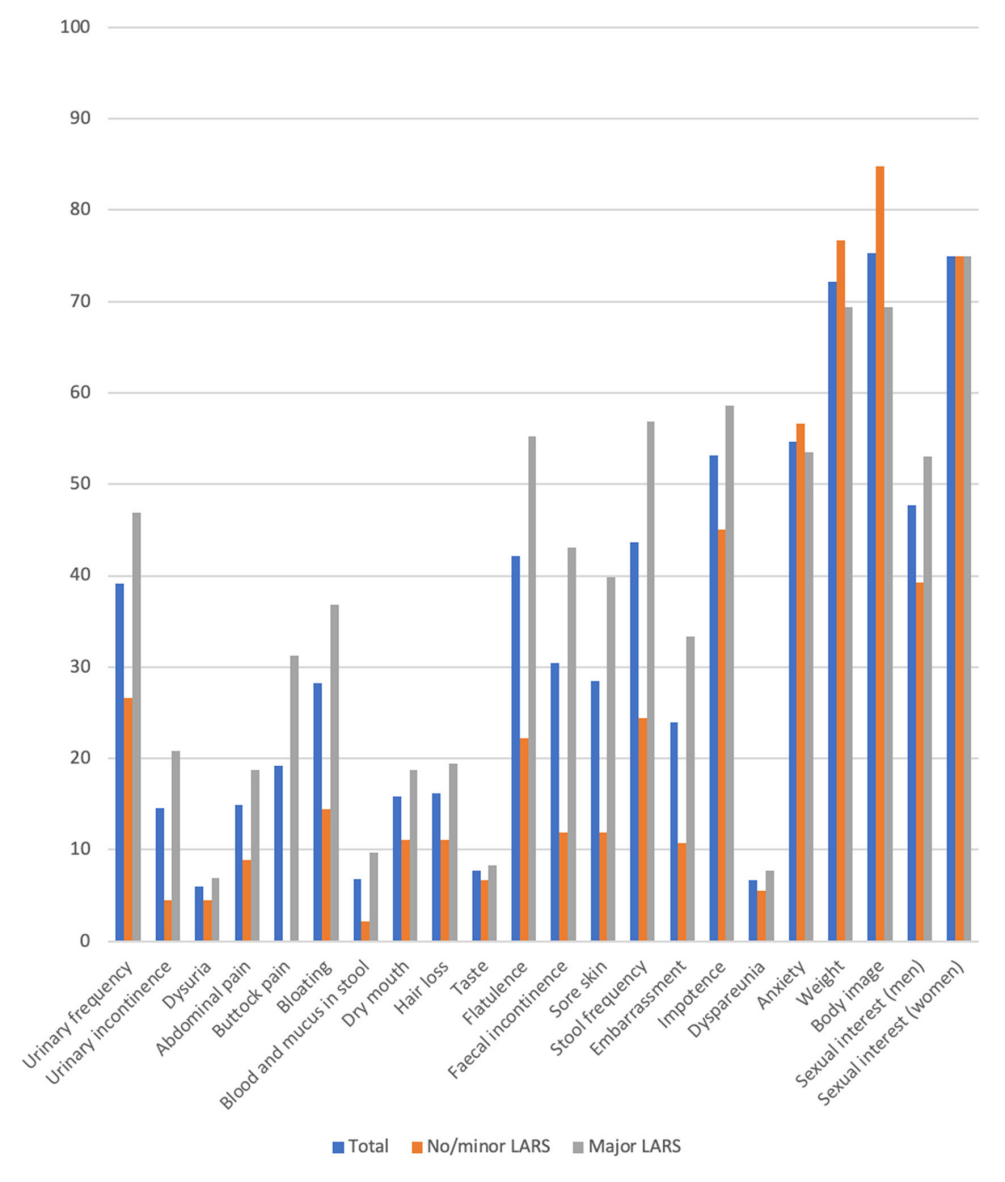
Results of the QLQ-CR 29 questionnaire comparing no/minor with major LARS (mean). Scoring according to the EORTC-scoring manual a high score in fuctional scales represents a high functional level wehreas a high score in symptom scales correlate with a high level of symptoms.

Focusing on the EORTC-C29 questionnaire, we observed significant differences between no/minor LARS and major LARS groups for urinary frequency (*p* = 0.003), urinary incontinence (*p* = 0.007), buttock pain (*p* < 0.001), bloating (*p* = 0.006), blood and mucus in stool (*p* = 0.011). In addition, significant differences were found for flatulence (*p* < 0.001), faecal incontinence (*p* < 0.001), stool frequency (*p* < 0.001) and sore skin (*p* = 0.003). Apart from the embarrassment (*p* = 0.008) and body image (*p* = 0.021), no further significant difference was reported for the sexual functioning (Figure 4).

The choice of the surgical approach (68 laparoscopic vs. 10 open) did not influence the mean LARS-score or the global health status. Furthermore, malignant nature of the rectal lesions had no significant impact on the postoperative mean global health status or the occurrence of a postoperative LARS (Table 3). The reported comorbidities such as cardiac or pulmonary diseases did not affect the postoperative LARS-score or the mean global health status. Regarding the Man-Whitney-U-test, the need of a life-long medication for any medical condition did not have a significant impact on the LARS (*p* = 0.906) or global health score (*p* = 0.812).

**Table 3:** Global health status and LARS-Score according to potential influencing factors.

	**Laparoscopic**	**Open/Conversion**	***p*** **-value**
Global Health status (mean ± SD)	67.89 ± 19.96	68.33 ± 24.15	0.712
LARS-score (mean ± SD)	29.22 ± 11.20	24.30 ± 14.06	0.216
	**Benign**	**Malignant**	***p*** **-value**
Global Health status (mean ± SD)	63.02 ± 23.95	69.22 ± 19.36	0.600
LARS-score (mean ± SD)	26.81 ± 11.82	29.05 ± 11.62	0.339

*Higher score in Global health status means better global health status*.

*Man-Whitney-U-test*.

Using the Kruskal-Wallis-test, we did not find a significant change in the LARS-score according to the time to follow up (*p* = 0.676). Additionally, no significant change was noticed in the follow-up-period in the global health status with a mean of 68 points (*p* = 0.465) (Table 4).

**Table 4:** LARS and global health status according to time to follow-up (months).

	**0–12** ***n*** **= 39**	**13–24** ***n*** **= 18**	**25–36** ***n*** **= 7**	**37–48** ***n*** **= 6**	**49–60** ***n*** **= 7**	**>60** ***n*** **= 1**	**Total** ***n*** **= 78**	***p*** **-value**
LARS (mean ± SD)	29.97 ± 9.94	30.61 ± 10.02	22.29 ± 16.93	24.67 ± 12.52	24.14 ± 16.87	37.00	28.59 ± 11.62	0.676^a^
Global Health status (mean ± SD)	69,87 ± 17.59	59.72 ± 21.82	76.19 ± 20.65	69.44 ± 19.48	69.05 ± 31.07	66.67	67.95 ± 20.37	0.465^a^

a *Kruskal-Wallis-Test*.

## Discussion

In our study, following a multimodal fail-safe perioperative protocol for rectal resections with end-to-end anastomosis and a temporary covering ileostomy, the patient-reported outcomes showed a high HRQOL with a global health status of 67.95 ± 20.37 points with no AL. The results signal the advantages of using a perioperative multimodal management protocol by adhering to surgical details in a structured fail-safe-protocol, thus reducing the postoperative AL rate with good functional outcomes after rectal resections and primary reconstruction.

Rectal resection causes the loss of specialized organ functions such as its reservoir function and the particularly the impairment of coordination between colonic movement, autonomic nerves and sphincter muscles. This complex interaction causes an increasing compartmentation following extended resection and deep anastomosis (34). This causes a rise in the LARS score following rectal dissection, resection and anastomosis, the surgical steps which explain the pathophysiology of LARS.

This has led to the development of different techniques for rectal reconstruction to construct a new reservoir using a pouch or a coloplasty. The advantages of the rectal reconstruction using a J-pouch have been shown by different international multicenter trials (35). Until today, clinical trials have not provided a concrete advantage of the J-pouch compared to other non-straight reconstruction modes (36, 37). On the same note, the study by Kupsch et al. did not report any notable difference between different non-straight-reconstruction modes for functional outcomes (38). Consequently, the recommendation by the German Guidelines for colorectal cancer is only a non-straight anastomosis, wherever achievable (26). A great majority of studies have shown better clinical outcomes within the first months after the rectal resection for non-straight anastomosis, but a reduced advantage in long-term follow-up (36, 39). Rybakov et al. compared straight vs. side-to-end anastomosis describing less bowel movements as the only benefit after 6 months (22). On the other hand, Lazorthes et al. showed functional improvements after rectal resections for 24 months (23). The long-term outcomes of a non-straight reconstruction after rectal resection remain unclear. According to our analysis, we could not find a major change of global health status or LARS-score over the follow-up-period (Table 4).

A relatively new surgical technique for rectal resection is the transanal total mesorectal excision (TaTME), which has shown comparable postoperative outcomes in the initial phase (40). Further studies showed more inconsistent results regarding this technique. De Simone et al. have described acceptable functional outcomes during the short-term-follow-up (41), while other studies have reported high rates of complications especially AL rates. These findings have provided an impetus to abandon the TaTME approach (42). Additionally, Bianco et al. have recently published a new technique for the rectal resection with an adopted pull-through anastomosis (43). This technique has demonstrated a comparable mean LARS-score after 12 and 36 months and a comparatively low leakage rate.

As a part of the multimodal fail-safe-concept used in our institution, a reconstruction using pouch or coloplasty or even side-to-end-anastomosis was not used to reduce the rate of AL. The studies evaluating the functional improvements by a pouch, side-to-end-reconstruction or coloplasty showed a relative high rate of AL. The meta-analysis presented by Hüttner et al. showed no significant differences in AL rate according to different reconstruction techniques with an AL rate ranging from 3.6% in J-pouch to 9.9% in another J-pouch-group (36). The AL rate for straight reconstruction was as high as 7.7%. In our cohort, 0% AL was recorded. Further refinements such as laparoscopic (18) or robotic surgery (44), pelvic intraoperative neuromonitoring (45), transanal total mesorectal excision (31) or fluorescence-guided imaging (46) may reduce the rates of AL and enhance the functional outcomes with or without a new reservoir made by pouch, side-to-end anastomosis or coloplasty.

### HRQOL

In our study, a major LARS occurred in 61.5% patients. This rate is in line with the internationally published data ranging from 41% (16) up to 52%, as reported by Juul et al. (13).

In 2019, Kupsch et al. compared the correlation between LARS and the QoL using the EORTC-questionnaires (5). In their study, the investigators found a reduced global quality of life, according to the EORTC-C30 questionnaire, in the group of patients with major LARS. This is also seen in our study whereas the measured global health status in patients with major LARS was higher [63 ± 20 vs. 56 ± 19 Kupsch et al. (5)].

### Limitations

As this is a retrospective analysis on the basis of a prospective database, no longitudinal comparison is achievable. Our data presents a median follow up of 1 year. A more longitudinal study design could establish the efficacy of the multi-modal fail-safe-protocol with substantial impact. Due to the small number of the answered questionnaires, the size of our study is small. Additionally, there are no internal or external control-groups with non-straight anastomosis, so an inter-group or pairwise comparison was not possible.

This study includes postoperative patients after surgery for benign and malignant rectal lesions. Even if there was no significant difference in the global QoL between both groups, this is a major study limitation, as the QoL and LARS could be influenced by neoadjuvant or adjuvant treatment, even if the surgery is performed following oncological criteria.

## Conclusion

Our study eludes that the functional outcomes following rectal resections with straight anastomosis are not worse than reported by reconstruction with J-pouch-, side-to-end anastomosis or coloplasty, even within the first 12 month of surgery. Despite our small study group, we emphasis that we did not record even a single AL following rectal resections and primary end-to-end anastomosis with temporary covering ileostomy. In conclusion, the straight anastomosis after rectal resection is an achievable procedure with a good functional outcome and a reduced leakage rate following the multimodal fail-safe-protocol.

## Data Availability Statement

The raw data supporting the conclusions of this article are available on request from the corresponding author.

## Ethics Statement

The studies involving human participants were reviewed and approved by Ethics Committee Medical Association Schleswig-Holstein Bismarckallee 8-12 23795 Bad Segeberg Germany. The patients/participants provided their written informed consent to participate in this study.

## Author Contributions

JH and VB collected and analyzed the data and drafted the manuscript. SK and HH drafted and reviewed the manuscript and made an impact on discussion. TS reviewed the manuscript and supervised the study. SG performed a scientific enrichment, linguistics manuscript drafting, and statistical analysis. All authors read and approved the final manuscript.

## Conflict of Interest

The authors declare that the research was conducted in the absence of any commercial or financial relationships that could be construed as a potential conflict of interest.

## Publisher's Note

All claims expressed in this article are solely those of the authors and do not necessarily represent those of their affiliated organizations, or those of the publisher, the editors and the reviewers. Any product that may be evaluated in this article, or claim that may be made by its manufacturer, is not guaranteed or endorsed by the publisher.
